# Editorial: Advancing Social Purpose in Organizations: An Interdisciplinary Perspective

**DOI:** 10.3389/fpsyg.2021.689734

**Published:** 2021-06-07

**Authors:** Monica Thiel, Gabriele Giorgi, Antonio Ariza-Montes, Nicola Mucci

**Affiliations:** ^1^University of International Business and Economics, Beijing, China; ^2^Department of Psychology, European University of Rome, Rome, Italy; ^3^Department of Management, Social Matters Research Group, Universidad Loyola Andalucía, Seville, Spain; ^4^Dipartimento di Medicina Sperimentale e Clinica, Università di Firenze, Firenze, Italy; ^5^Direzione Sanitaria - Azienda Ospedaliero, Universitaria Careggi, Firenze, Italy

**Keywords:** social responsibility, sustainability, corporate responsibility, stakeholders, business purpose

Currently, there are many skeptics questioning the credibility of the Business Roundtables's pledge and commitment to build and deliver transparent socially responsible corporate governance that delivers value for all stakeholders' future success in business, communities, and country. Rodríguez-Vilá and Bharadwaj suggest that changing course on a firm's social purpose is difficult and ill-advised because success depends on the legitimacy of the brand's claim. Consequently, inconsistent social purpose claims may raise stakeholder doubts about the firm's integrity or commitment to social purpose. For instance, “social responsibility could be employed for stakeholder social governing power and economic benefits, rather than societal good” (Thiel, p. 1). Alternatively, Donaldson and Walsh propose there is no theory of the firm that can serve us well when we attempt to understand the purpose or place of business in society. The authors develop the beginnings of a theory of business that is both empirical and normative to highlight collective value. Clearly, social purpose in organizations is an underdeveloped topic. This Research Topic aims to advance social purpose in organizations to better understand how to connect the collective value link between the organization and society. The 13 articles in this Research Topic contribute to the literature for advancing social purpose in organizations through research studies from different sectors, industries, countries, and cultures. These studies are expected to deepen interdisciplinary knowledge on social purpose in organizations and lay a foundation for interested scholars to undertake in their future inquiries.

The first article in the Research Topic authored by Zhang and Sun argue that Chinese recent graduates' zero-sum construal of workplace success is a key factor influencing his or her initial workplace adaptability and initial work role behavior due to China's current and continuous economic development. The authors' findings indicate that social purpose in Chinese organizations could be improved by taking actions to enhance an individual's zero-sum construal of workplace success and prevention focus level. This will require human resource management to provide detailed information about the job-search market, and presenting and emphasizing the exact and specific data of loss or cost. Man et al. propose an identity-blind diversity management strategy in the second article that could yield positive organizational practices and outcomes toward the treatment of all employees in the workplace through a continuous view of disability that defines everyone with a certain level of disability ranging from zero to a high level of disability severity. Gan et al. research study in article three provides additional insights between public service motivation (PSM) and employee turnover intention to verify the causal effect of PSM on employees' turnover intention in Asian countries within a Confucian culture. The authors' findings reveal that public employees' PSM had no direct effect on their turnover intention when job satisfaction and organizational commitment were considered simultaneously.  Li et al. research findings in the fourth article indicate migrant workers in China holding dual-identity may have decreased emotional exhaustion because of higher perceptions of internal corporate social responsibility (CSR) efforts, and increased emotional exhaustion due to higher perceptions of job complexity that could be weakened by employees' shared perceptions of human resource practice strengths.

The authors Calderón et al. in the fifth article develop a second-generation definition of independence based on a positive approximation to CSR by integrating an Aristotelian perspective of virtue ethics with the best practices of corporate governance. Independence is defined as a virtue guided by practical wisdom with autonomy and autarky to enable a person to act with integrity, fairness, and truthfulness. Thus, independence is associated with an honest disposition to serve through corporate governance. Saiz-Álvarez et al. contribute to scarce literature on B-Corporations in the sixth article through examination of an organization's social purpose as a fundamental part of the production structure, rather than a consequence of a successful firm after profit and capital accumulation. In the seventh article authored by Gu et al., the authors contribute toward developing the concept of employees' sense of gain to confirm that employee satisfaction is a main source of a firm's competitive advantage. The authors' study confirms employees' perceived organizational support and their sense of gain will become enhanced. Consequently, employees will contribute more positively to the firm. Galuppo et al. provide insights into how to develop a more holistic and critical approach to sustainable tourism through education and communication in article number eight. Specifically, new educational approaches are required to move tourists from spectators or consumers of the natural environment, culture, social, and economic resources of a destination toward higher awareness of their power and responsibilities in co-generating a sustainable tourism experience. The authors of the ninth article, Robinson et al. conducted an interdisciplinary research study perspective to understand how obesity prevention and population nutrition are considered by Australian institutional investors engaged in responsible investment. The study was the first to review major institutional investors in Australia on their approaches to incorporating environment, social, and governance issues related to obesity and nutrition within their decision making. Although some institutional investors in Australia recognize the potential importance of incorporating obesity and population nutrition issues into decision-making processes, the extent to how these considerations translate into investment decisions and their impact on firms in the food sector warrant further exploration.

In article number 10, Fregnan et al. analyze an organizational case study to determine how human resource management as a social practice could be embedded within specific contexts through research questions. The findings highlight the connection between new technologies and human resource management in managing differences in technological cognitive frames with different internal stakeholders. Through a systematic review of the literature on corporate sustainability paradox, Luo et al. findings in article number 11 reveal (a) environmental and cognitive factors manifest tensions arising from a sustainability paradox and (b) the proactive strategy is more extensively studied in the current literature. In practice, the results imply that organizations should manage the corporate sustainability paradox by understanding the paradox and its equilibrium stages. Carroll et al. draw on adaptive governance literature in article number 12 to frame governance challenges and recommend five paradoxes requiring collective navigation. The authors engage with Indigenous scholarship through a series of recommendations on how to navigate paradoxes for when building governance practice in innovative social purpose initiatives. The authors of article number 13, namely Lambrechts et al. study contributes to the existing literature on social entrepreneurship by providing new insights into the role of empathy and life events. The study reveals that empathic involvement is an important driver for social entrepreneurs. About half of the social entrepreneurs interviewed by the authors describe their motivations in a way that aligns with empathy. However, it is not always clear whether a life event increases empathy, but it can be a reason to do things differently. Clearly, there are many differing ways to advance social purpose in organizations. The editors hope that this Research Topic will stimulate further research from readers that find social purpose in organizations important to further explore and develop within the organizational psychology literature.

## Author Contributions

All authors listed have made a substantial, direct and intellectual contribution to the work, and approved it for publication.

## Conflict of Interest

The authors declare that the research was conducted in the absence of any commercial or financial relationships that could be construed as a potential conflict of interest.
